# T‐cell immunoglobulin mucin 3 blockade drives an antitumor immune response in head and neck cancer

**DOI:** 10.1002/1878-0261.12029

**Published:** 2017-01-19

**Authors:** Jian‐Feng Liu, Si‐Rui Ma, Liang Mao, Lin‐Lin Bu, Guang‐Tao Yu, Yi‐Cun Li, Cong‐Fa Huang, Wei‐Wei Deng, Ashok B. Kulkarni, Wen‐Feng Zhang, Zhi‐Jun Sun

**Affiliations:** ^1^ The State Key Laboratory Breeding Base of Basic Science of Stomatology & Key Laboratory of Oral Biomedicine Ministry of Education School and Hospital of Stomatology Wuhan University China; ^2^ Department of Oral Maxillofacial‐Head Neck Oncology School and Hospital of Stomatology Wuhan University China; ^3^ Functional Genomics Section Laboratory of Cell and Developmental Biology National Institute of Dental and Craniofacial Research National Institutes of Health Bethesda MD USA

**Keywords:** effector T cells, head and neck squamous cell carcinoma, immunotherapy, myeloid‐derived suppressor cells, T‐cell immunoglobulin mucin 3

## Abstract

T‐cell immunoglobulin mucin 3 (TIM3) contributes to immune suppression during progression of many cancers, but the precise role of TIM3 in head and neck squamous cell carcinoma (HNSCC) is not clearly understood. In this study, we report that TIM3 expression was significantly up‐regulated in patients with HNSCC and associated with lymph node metastasis. Additionally, TIM3 expression was increased in patients with recurrent HNSCC and patients with preradiotherapy or prechemotherapy. We also characterized CD8^+^ T cells and CD11b^+^ CD33^+^ myeloid‐derived suppressor cells (MDSCs) in human HNSCC, and found that their expression was positively correlated with TIM3 expression. To determine the underlying mechanism of TIM3 in immune response during HNSCC progression, we utilized the *Tgfbr1/Pten* 2cKO HNSCC mouse model with TIM3 overexpression. Treatment with anti‐TIM3 monoclonal antibody effectively suppressed tumor growth through restoring effector T‐cell function by targeting CD4^+^ TIM3^+^ cells and CD8^+^ TIM3^+^ cells and decreasing MDSCs. Our findings demonstrate TIM3 expression in patients with HNSCC and suggest anti‐TIM3 immunotherapy as a novel therapeutic approach for effective treatment of HNSCC.

**Abbreviations**
HNSCC: head and neck squamous cell carcinoma
HPV: human papillomavirus
LN: lymph node
mAb: monoclonal antibody
MDSCs: myeloid‐derived suppressor cells
TIM3: T‐cell immunoglobulin mucin 3
TPF: docetaxel, cisplatin, and fluorouracil
WT: wild‐type

## 1 Introduction

Head and neck squamous cell carcinoma (HNSCC) is the sixth most common cancer worldwide (Siegel *et al*., 2014). Tobacco use and alcohol consumption are the main important risk factors for HNSCC (Argiris *et al*., 2008). Human papillomavirus (HPV) infection is recognized as another increasing high‐risk factor for HNSCC (Marur *et al*., 2010). Although treatment regimen with appropriate chemotherapy, surgery, and radiation therapy has improved clinical outcomes in recent decades, survival rate of patients with HNSCC has not markedly improved because of local recurrences, distant metastasis, and secondary primary tumors (Leemans *et al*., 2011).

HNSCC is an immunosuppressive malignancy, with lymphocyte deficiencies (Baruah *et al*., 2012; Whiteside, 2005), impaired immune effector cells (Bauernhofer *et al*., 2003; De Costa *et al*., 2012; Kloss *et al*., 2015), and poor antigen presentation (Ferris *et al*., 2006; Lopez‐Albaitero *et al*., 2006). In addition, myeloid‐derived suppressor cells (MDSCs), which often characterized as Lin^−^CD33^+^CD11b^+^HLA^−^DR^−^ in humans (Ugel *et al*., 2009), have been reported to be linked to HNSCC tumor progression (Strauss *et al*., 2007; Weed *et al*., 2015). MDSCs have been observed in a number of mouse tumor models and identified by the presence of CD11b^+^Gr‐1^+^ cells (Gabrilovich and Nagaraj, 2009). In recent years, with better understanding of immune dysfunction in the evolution of HNSCC, immunotherapy that targets ‘immune checkpoint’ is considered as a promising treatment option to improve outcomes and survival for patients (Ferris, 2015).

T‐cell immunoglobulin mucin 3 (TIM3), a member of TIM family, is an immune checkpoint molecule (Sharma and Allison, 2015). It is expressed on T‐helper 1 cells and dendritic cells, CD8^+^ T cells, and other lymphocyte subsets (Anderson *et al*., 2007; Freeman *et al*., 2010; Monney *et al*., 2002). Binding with galectin‐9, TIM3 induced Th1 cell death, suggesting its function in negative regulation of Th1 response (Zhu *et al*., 2005). A recent research also demonstrated that interactions between CEACAM1 and TIM3 determine the function of TIM3 (Huang *et al*., 2015). Emerging evidences demonstrated TIM3 as an important regulator of CD8^+^ T‐cell exhaustion in cancer (Fourcade *et al*., 2010). The exhaustion of T cells induces T‐cell dysfunction in immune response and thus prevents optimal control of tumors. TIM3 blockade with anti‐TIM3 monoclonal antibody (mAb) was found to increase IFN‐γ‐mediated T cells (Sabatos *et al*., 2003).

In the present study, we investigated the expression of TIM3 in human HNSCC and determined its role in HNSCC progression. Taking advantage of immunocompetent transgenic mouse HNSCC models, we observed the progression of tumor formation and its correlation with the changes in the immune cells in tumor microenvironment as well as in peripheral environment.

## 2 Materials and methods

### 2.1 Ethics statement, patients’ specimens, and human HNSCC tissue microarray

This study was authorized by the School and Hospital of Stomatology of Wuhan University Medical Ethics Committee, and informed consent was accepted from the patients before the surgery. All the HNSCC patients’ tissues were collected from the Hospital of Stomatology of Wuhan University. The clinical stage of HNSCC was classified according to the guidelines of the International Union Against Cancer (UICC, Sobin *et al*., 2002), and histological grade was affirmed based on the classification scheme of the World Health Organization. Human HNSCC tissue microarrays were constructed and used for immunohistochemistry staining, including 27 normal mucosa, 43 dysplasia (Dys), 122 primary HNSCC, eight recurrent HNSCC, 12 HNSCC with preoperation radiotherapy, and 11 HNSCC with preoperation chemotherapy (Table S1).

### 2.2 Immunohistochemistry

The HNSCC tissue array sections were hydrated, and antigen retrieval was performed. After blocked with 2.5% bovine serum album in PBS buffer for 1 h at 37 °C, the tumor sections were stained with the antibody for TIM3 (Cell Signaling Technology, Danvers, MA, USA), CD8 (ZSGB‐BIO, Beijing, China), CD11b (Abcam, Cambridge, UK), and CD33 (ZSGB‐BIO, Beijing, China) at 4 °C overnight. The second day, the sections were incubated with a secondary biotinylated immunoglobulin G antibody solution and an avidin/biotin/peroxidase reagent. Finally, the sections were counterstained with Mayer's hematoxylin (Invitrogen, Waltham, MA, USA). Staining with isotype‐matched IgG was used as negative controls.

### 2.3 Spontaneous HNSCC mouse models

All experiments were conducted in accordance with guidelines of the Institutional Animal Care and Use Committee of the Wuhan University. The squamous epithelial tissue‐specific and time‐inducible combined *Tgfbr1/Pten*‐knockout mice (*K14‐Cre* ^ERtam+/−^;*Tgfbr1* ^flox/flox^; *Pten* ^flox/flox^) were maintained and genotyped as previously described (Bian *et al*., 2012; Sun *et al*., 2012). The *Tgfbr1/Pten* 2cKO mice and their vehicles (*Tgfbr1* ^flox/flox^; *Pten* ^flox/flox^) came from the same litter and with same mixed genetic background of C57BL/6; FVBN; CD1; 129. Oral gavage of tamoxifen was applied for five consecutive days to knock out *Tgfbr1/Pten* in oral and head neck epithelia. The procedure of tamoxifen application has been previously described (Bian *et al*., 2012; Sun *et al*., 2012). All animal studies were carried out in accordance with the NIH guidelines in the SPF Animal Laboratory of Wuhan University School & Hospital of Stomatology and approved by the Animal Care and Use Committee of Wuhan University.

### 2.4 TIM3 antibody treatment

Rat anti‐TIM3 monoclonal antibody (*InVivo*MAb clone RMT3‐23) and rat IgG2a isotype control (*InVivo*MAb clone 2A3) were purchased from BioXCell (West Lebanon, NH, USA). Only 4‐ to 8‐week‐old male and female *Tgfbr1/Pten* 2cKO mice were used for this study. For anti‐TIM3 monoclonal antibody (mAb) therapy, 2 weeks after the last dose of oral tamoxifen gavage, the mice were randomized into an isotype control (*n* = 6) group or an anti‐TIM3 group (*n* = 6). Wild‐type (WT) mice with same mixed genetic background were used for blank control. Mice were treated prophylactically with isotype IgG2a or intraperitoneally (100 μg intraperitoneal) with anti‐TIM3 on days 12, 13, and 14 and then weekly for the rest of the treatment. Tumor size of *Tgfbr1/Pten* 2cKO mice was measured and photographed every other day. In the end, the mice were euthanized and the tumors were fixed in paraffin for the following IHC analysis.

### 2.5 Flow cytometry

The single‐cell suspensions from spleens, draining lymph node (LN), blood, and tumor from WT and *Tgfbr1/Pten* 2cKO mice were processed according to a standardized protocol (Trellakis *et al*., 2013). Tumors from *Tgfbr1/Pten* 2cKO mice were excised and digested and processed using a gentle Macs dissociator and a murine tumor dissociation kit (Miltenyi Biotec, Bergisch Gladbach, Germany). Flow cytometry analysis of cells was performed by flowjo (Tree Star, Ashland, OR, USA), and cells were gated by surface markers and negative controls (Yu *et al*., 2016). Death cells were excluded by staining with 7AAD (Invitrogen). The following anti‐mouse antibodies were used for fluorescence staining: FITC‐conjugated CD4, CD8, and CD11b; PE‐conjugated TIM3 and Gr‐1 (all from Becton Dickinson, Mountain View, CA, USA). Cells stained with isotype‐matched IgG were used as negative controls (eBioscience, San Diego, CA, USA).

### 2.6 Immunofluorescence

Tumors from mice were excised and fixed for sections. Tumor sections were hydrated, and antigen retrieval was performed. After blocked with 2.5% bovine serum album in PBS buffer for 1 hour at 37 °C, the tumor sections were stained with the primary antibody at 4 °C overnight, followed by incubation with fluorochrome‐conjugated secondary antibodies (Alexa 594 anti‐rabbit and Alexa 488 anti‐mouse; Invitrogen) and mounting in Vectashield with 4′, 6‐diamidino‐2‐phenylindole (Vector Laboratories; Yu *et al*., 2016). Fluorescence images were captured using a CLSM‐310, Zeiss fluorescence microscope (Zeiss, Oberkochen, Germany). Cells stained with isotype‐matched IgG were used as negative controls.

### 2.7 Western blot

Tumor sections from HNSCC mouse model were carefully dissected. A total amount of 30 μg protein from each sample was denatured and then subjected to 12% SDS/polyacrylamide gel electrophoresis followed by transfer onto polyvinylidene fluoride membranes (Millipore Corporation, Billerica, MA, USA). Next, the blots were stained using an enhanced chemiluminescence detection kit (West Pico, Thermo Fisher, Waltham, MA, USA) (Yu *et al*., 2016). The following antibody was used for western blot analysis: CXCL1 (GeneTex, Irvine, CA, USA). β‐Actin was used as a loading control.

### 2.8 Scoring system, hierarchical clustering

Tissue array slices were scanned using an Aperio ScanScope CS scanner (Vista, CA, USA) with background subtraction and quantified with aperio quantification software (version 9.1) for membrane, nuclear, or pixel analyses. An area of interest was selected either in the epithelial or in the cancerous area for scanning and quantification. The histoscore of membrane and nuclear staining was calculated as a percentage of different positive cells using the formula (3+) × 3 + (2+) × 2 + (1+) × 1. Histoscore of pixel quantification was calculated as total intensity/total cell number (Sun *et al*., 2012). Histoscores were translated to scaled values centered at zero in Microsoft Excel, and the hierarchical analysis was performed by the cluster 3.0 (Eisen *et al*., 1998) and java treeview 1.0.5 (Saldanha, 2004).

### 2.9 Statistical analysis

Statistical analysis was performed with statistical package graphpad prism 5.01 (GraphPad Software, Inc., La Jolla, CA, USA). One‐way ANOVA followed by the *post hoc* Tukey's multiple comparison tests and unpaired *t*‐test was used to analyze the differences in IHC staining and positive cells among each group. Two‐tailed Pearson's correlation coefficient was used to determine the correlation between expression of TIM3 and CD8, CD33, and CD11b after confirmation of the sample using a Gaussian distribution. The Kaplan–Meier curve was used to analyze survival of patients with HNSCC, while the log‐rank test was used to detect the differences in overall survival. Mean values ± SEM with *P* < 0.05 was considered statistically significant.

## 3 Results

### 3.1 TIM3 expression is elevated in patients with HNSCC

**Figure 1:**
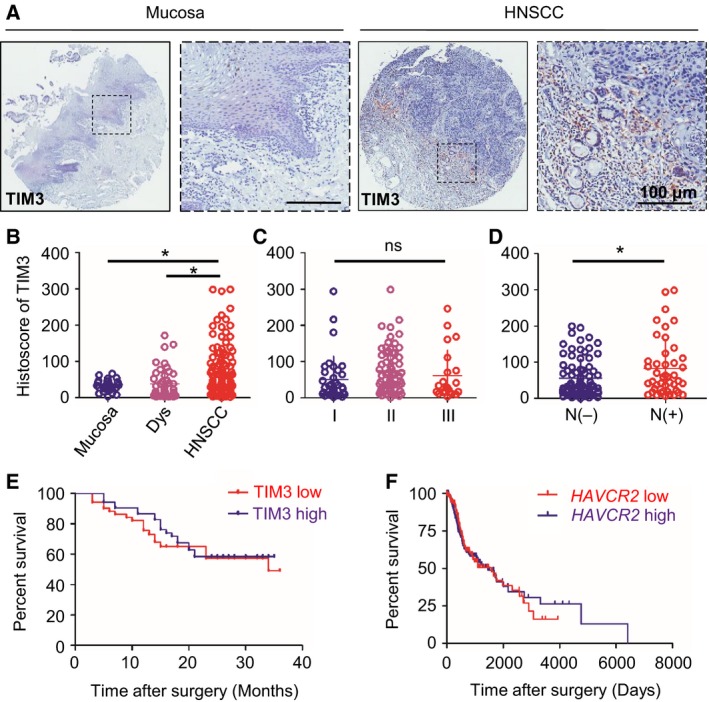
TIM3 expression in human head and neck squamous cell carcinoma(HNSCC)tissue. (A) Representative pictures of TIM3 expression in normal mucosa (left panel) and HNSCC (right panel) by immunohistochemical (IHC) staining. (B) Quantification of histoscore of TIM3 expression in normal mucosa (*n* = 27), epithelial dysplasia (Dys, *n* = 43), and HNSCC (*n* = 122) (*P <* *0.05; one‐way ANOVA followed by *post hoc* Tukey's analysis). (C) TIM3 expression in patients with different pathological grades. (D) TIM3 expression in patients with lymph node metastasis (N−) (*n* = 78) or without lymph node metastasis (N+) (*n* = 44) (*P <* *0.05; *t*‐test). (E) Survival analysis based on TIM3 expression using Kaplan–Meier curve. Patients were divided into two groups by the median expression of TIM3. The difference between patients with high TIM3 expression (*n* = 51) and low TIM3 expression (*n* = 52) did not reach statistical significance (*P *=* *0.5143). (F) Survival analysis based on *HAVCR2* (gene encoding TIM3) expression using Kaplan–Meier curve from TCGA database. Patients were divided into two groups by the median expression of *HAVCR2*. The difference between patients with high *HAVCR2* expression (*n* = 283) and low *HAVCR2* expression (*n* = 296) did not reach statistical significance (*P *=* *0.9365).

To determine the TIM3 expression levels in human HNSCC, we checked the Oncomine database (www.oncomine.org) (Rhodes *et al*., 2007). In a meta‐analysis of nine datasets of head and neck cancer gene expression profiling, *HAVCR2* (gene encoding TIM3) DNA copy number and mRNA expression were both significantly increased in HNSCC as compared with the controls (*P* < 0.001, Fig. S1A,B). We examined TIM3 expression in patients with HNSCC using tissue microarray. An elevated TIM3 expression was consistently found in inflammatory cells of the cancerous tissue (Fig. 1A), and quantification analysis showed the significant up‐regulation of TIM3 in HNSCC (*n *= 122) as compared with dysplasia (*n* = 43) and normal mucosa (*n* = 27) (*P < *0.05, Fig. 1B). Analysis of clinical data suggested that TIM3 expression was not correlated with pathological grades (Fig. 1C). However, results showed that increased TIM3 expression was correlated with LN metastasis (*P *< 0.05, Fig. 1D). Then, we assessed the prognostic implications of TIM3 in patients with HNSCC. One hundred and three patients with primary HNSCC were selected and divided into TIM3 high (*n* = 51) and TIM3 low (*n* = 52) groups. The Kaplan–Meier curves indicated that TIM3 expression was not related to the survival of patients with HNSCC (Fig. 1E). Interestingly, data obtained from TCGA database (cancergenome.nih.gov) also demonstrated that there is no correlation between *HAVCR2* (gene encoding TIM3) expression and survival of patients with HNSCC (Fig. 1F).

### 3.2 TIM3 expression in recurrent HNSCC, HNSCC with preradiotherapy, and HNSCC with pre‐TPF chemotherapy

**Figure 2:**
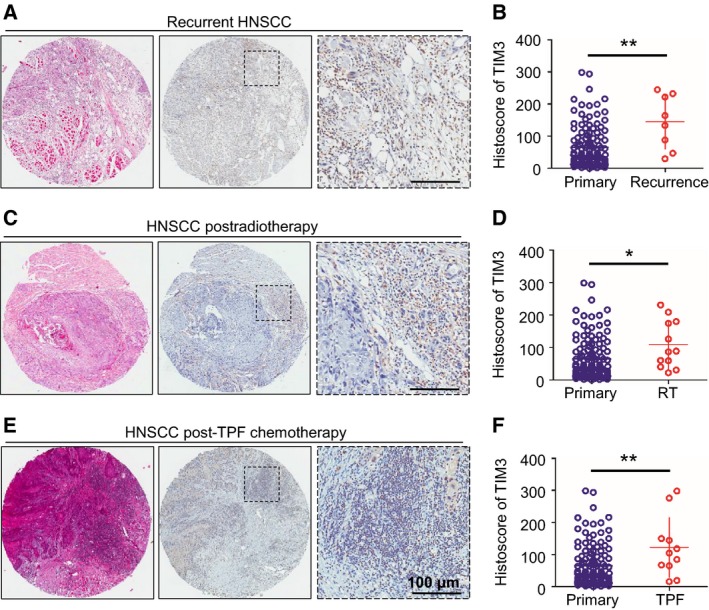
TIM3 expression in recurrent HNSCC, NSCC postradiotherapy, or post‐TPF chemotherapy. Representative pictures of hematoxylin/eosin staining and IHC staining of TIM3 in recurrent HNSCC (A), HNSCC postradiotherapy (C), and HNSCC post‐TPF chemotherapy (E), and quantification of histoscore showed the increased TIM3 expression in recurrent HNSCC (B) (*n* = 8), HNSCC postradiotherapy (D) (RT,* n *= 12), and HNSCC post‐TPF chemotherapy (F) (TPF,* n* = 11) as compared with that in primary HNSCC (*n* = 122) (*P <* *0.05, **P <* *0.01, *t‐*test).

In order to carry out comprehensive analysis of TIM3 expression in patients with HNSCC at different stages of cancer progression and therapeutic regimen, we analyzed TIM3 expression in recurrent HNSCC, HNSCC postradiotherapy, and HNSCC post‐TPF chemotherapy (cisplatin, docetaxel, and fluorouracil). Representative images of hematoxylin/eosin staining and TIM3 immunostaining (IHC) are shown in Fig. 2. The quantification of IHC staining demonstrated that TIM3 expression was significantly increased in recurrent HNSCC (*P *< 0.01, *n* = 8, Fig. 2B) and HNSCC with preradiotherapy (*P *< 0.05, *n* = 12, Fig. 2D) or pre‐TPF chemotherapy (*P *< 0.01, *n* = 11, Fig. 2F) as compared with primary HNSCC (*n* = 122). In summary, these results indicated that increased TIM3 expression is correlated with recurrent HNSCC, HNSCC with preradiotherapy, and HNSCC with pre‐TPF chemotherapy.

### 3.3 TIM3 expression correlates with CD8, CD11b, and CD33 in human HNSCC tissue

**Figure 3:**
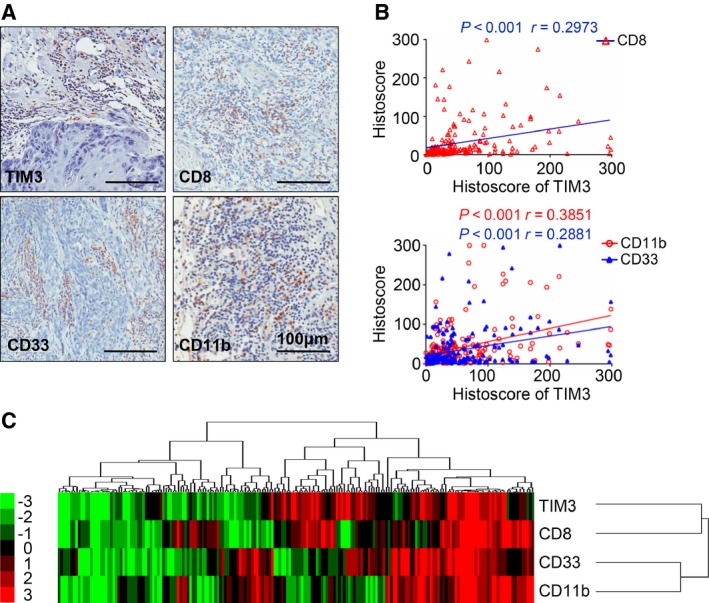
TIM3 expression is correlated with CD8, CD33, and CD11b in human HNSCC tissue array. (A) Representative pictures of IHC staining of CD8, CD11b, and CD33 in human HNSCC. Scale bar, 100 μm. (B) Correlation of TIM3 with CD8 (*P *<* *0.001, *r* = 0.2973), CD11b (*P *<* *0.001, *r *= 0.3851), and CD33 (*P *<* *0.001, *r* = 0.2881). (C) Hierarchical clustering presents the protein expression correlation between TIM3, CD8, CD11b, and CD33 in human HNSCC tissue microarray.

To better understand the immune status of patients with HNSCC, we also investigated the markers of effector T cells and MDSCs in tissue microarray, including CD8, CD11b, and CD33. As shown in the representative images (Fig. 3A), these markers were mainly expressed on inflammatory cells. Interestingly, by analyzing the quantification of IHC staining and performing the Spearman's rank correlation coefficient test and linear tendency test, we found that TIM3 expression in HNSCC was significantly correlated with CD8 (*P *<* *0.001, *r* = 0.3126), CD11b (*P *<* *0.001, *r* = 0.3892), and CD33 (*P *<* *0.001, *r* = 0.3089, Fig. 3B). The cluster results show the IHC scores of TIM3, CD8, CD11b, and CD33 of each patient, which also indicates the close association between TIM3 and CD8, CD11b, and CD33 (Fig. 3C). These findings imply that TIM3 expression is correlated with CD8^+^ T cells and MDSCs in human HNSCC.

### 3.4 Elevation of TIM3 expression and reduction in effector T cells in the *Tgfbr1/Pten* 2cKO mouse HNSCC model

**Figure 4:**
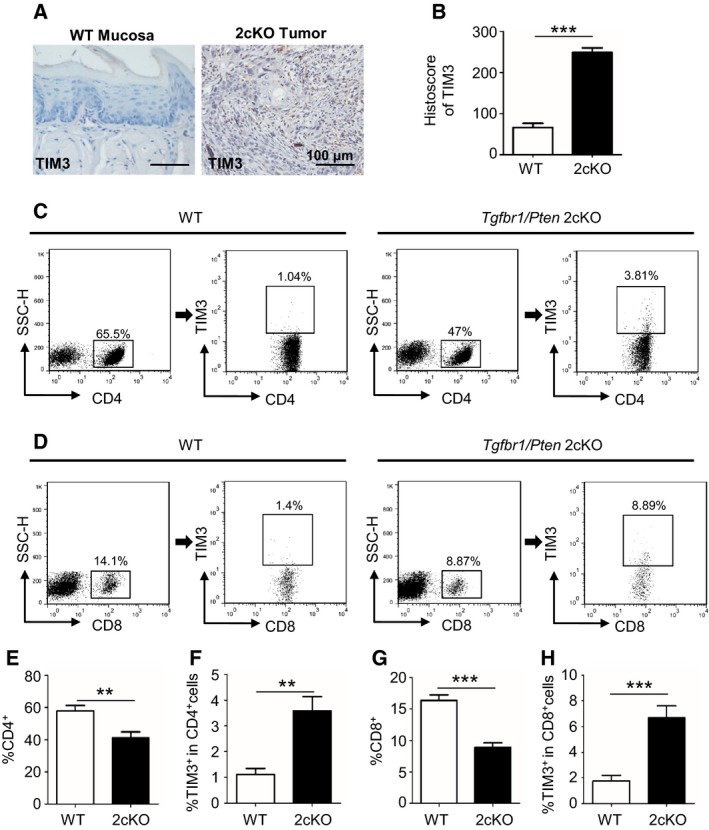
TIM3 expression is elevated, and effector T cells are reduced in the *Tgfbr1/Pten* 2cKO mouse HNSCC model. (A) Representative IHC staining of TIM3 in mucosa of wild‐type mice (left) and tumor of *Tgfbr1/Pten* 2cKO mice (right). (B) Histoscore of TIM3 expression in each group of mice (mean ± SEM,* n *=* *6 mice, respectively, *t*‐test, ***P <* *0.001). (C) The representative FACS plots of CD4^+^ cells and TIM3 expression on CD4^+^ cells from draining lymph nodes (LN) of WT mice and *Tgfbr1/Pten* 2cKO mice. (D) The representative FACS plots of CD8^+^ cells and TIM3 expression on CD8^+^ cells from LN of each group. The quantification of CD4^+^ cells ratio (E) and TIM3^+^ CD4^+^ cells ratio (F) in 2cKO tumor‐bearing mice as compared with wild‐type (WT) group. The quantification of CD8^+^ ratio (G) and TIM3^+^ CD8^+^ ratio (H) in the two groups (mean ± SEM,* n *=* *6 mice, respectively, *t*‐test, *P <* *0.05, **P <* *0.01, ***P <* *0.001).

As transforming growth factor‐β (TGF‐β) and PTEN/PI3K/Akt pathways are among the most frequently altered signaling routes in the process of HNSCC development, *Pten* deletion in the mice head and neck epithelia gives rise to the activation of PI3K/Akt pathway, and loss of *Tgfbr1* in the head and neck epithelia enhances paracrine effect of TGF‐β on the tumor stroma. *Pten‐* and *Tgfbr1*‐deficient mice develop full‐penetrance HNSCC, and this mouse model is immunocompetent (Bian *et al*., 2012). Given the multiple molecular alternation and pathology of the 2cKO mice tumor resembling human HNSCC, the 2cKO mouse model is suitable for studying the development of cancer and strategies for the prevention of HNSCC, especially for immunotherapy. To determine TIM3 expression in the HNSCC mouse model, we carried out the IHC staining of TIM3 and found that TIM3 expression was elevated in tumors of *Tgfbr1/Pten* 2cKO mice (Fig. 4A,B). Furthermore, we analyzed the population of effector T cells, CD4^+^ and CD8^+^ T cells from draining LNs in WT mice and *Tgfbr1/Pten* 2cKO mice (Fig. 4C,D). The results of these studies demonstrated that the CD4^+^ and CD8^+^ T cells were reduced in *Tgfbr1/Pten* 2cKO mice (Fig. 4E,G). Interestingly, the TIM3 expression on CD4^+^ or CD8^+^ T cells was up‐regulated (Fig. 4F,H). These findings suggest that TIM3 may induce the reduction in effector T cells in HNSCC mice, and provide the basis for the development of anti‐TIM3 treatment.

### 3.5 Anti‐TIM3 therapy suppresses tumor growth in HNSCC mouse model

**Figure 5:**
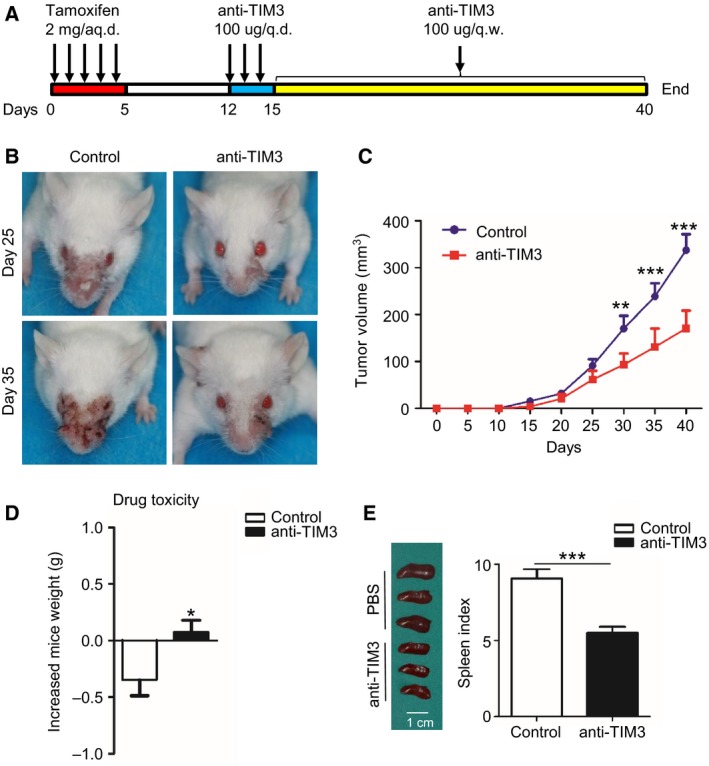
Anti‐TIM3 therapy suppresses tumor growth in the *Tgfbr1/Pten* 2cKO HNSCC mouse model. (A) Schematic representation of procedure that induces tumor formation and anti‐TIM3 therapy. (B) Representative photographs of mice with head and neck tumor after treatment with IgG or anti‐TIM3 at days 25 and 35. (C) Tumor volumes were measured and recorded every five days (mean ± SEM,* n *=* *6 mice, respectively, *t‐*test, **P <* *0.01, ***P <* *0.001). (D) Drug toxicity was determined by gain of body weight of mice in each group (mean ± SEM,* n *=* *6 mice, respectively, *t*‐test, *P <* *0.05). (E) Representative photographs (left) show the different size of spleen in mice treated with or without anti‐TIM3 mAb, and spleen index (right) demonstrated that anti‐TIM3 suppresses compensatory growth of spleen in HNSCC mouse model (mean ± SEM,* n *=* *6 mice, respectively, *t*‐test, ***P <* *0.001).

To evaluate the effect of anti‐TIM3 therapy on the spontaneous tumor growth, we employed the chemopreventive experiment by utilizing the *Tgfbr1/Pten* 2cKO mice. After tamoxifen induction of tumor formation, mice were initially treated with IgG or anti‐TIM3 mAb on days 12, 13, and 14 and then weekly for the rest of the treatment (Fig. 5A**)**. The tumor‐bearing mice treated with IgG demonstrate rapid tumor growth, while mice treated with anti‐TIM3 mAb showed a decreased rate of tumor growth as seen from tumor volumes in anti‐TIM3 group, which was significantly smaller than control group on days 30, 35, and 40 (Fig. 5B,C). These results suggest that anti‐TIM3 therapy will suppress tumor growth in immunocompetent HNSCC mice. The use of anti‐TIM3 mAb did not cause additional toxic and side effect, albeit this treatment showed moderate gain of weight, as judged by the gain of body weight in the treated mice as compared to the control group (*n* = 6, respectively, *P *< 0.05, Fig. 5D). Additionally, the spleen size of control group showed compensatory hypertrophy as compared with that of the anti‐TIM3 group (Fig. 5E), which indicates the better immune status of the mice treated with anti‐TIM3 mAb.

### 3.6 Blockade of TIM3 restores effector T cells by modulating TIM3 expression on CD4^+^ or CD8^+^ T cells and decreasing MDSCs in HNSCC mouse model

**Figure 6:**
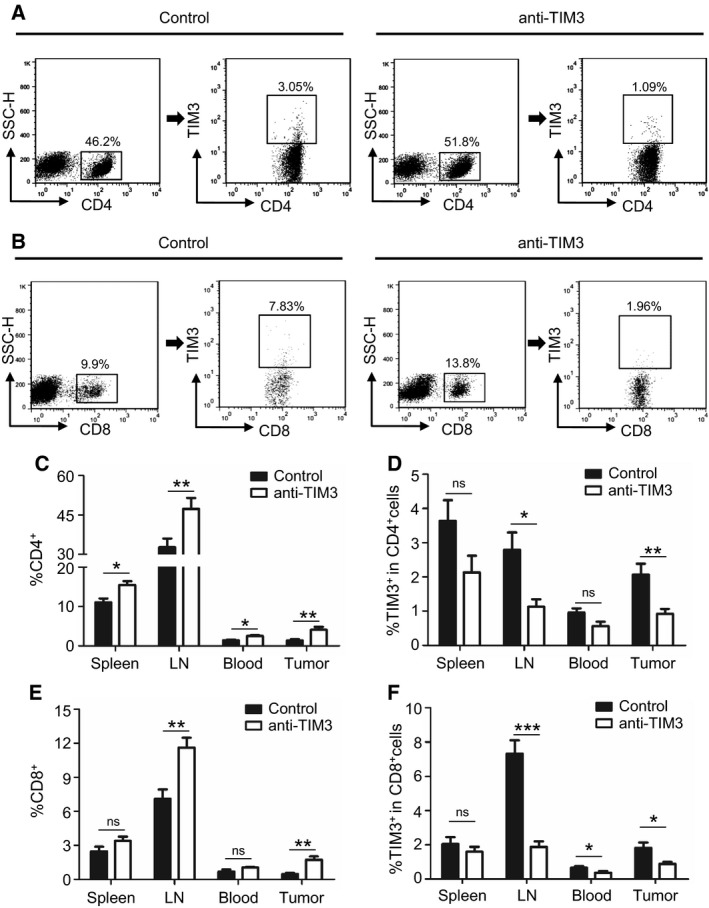
Blockade of TIM3 increases effector T cells in HNSCC mouse model through modulating the CD4^+^ TIM3^+^ cells and CD8^+^ TIM3^+^ cells. (A) The representative FACS plots of CD4^+^ cells and TIM3 expression on CD4^+^ cells in mice treated with either IgG or anti‐TIM3 mAb. (B) The representative FACS plots of CD8^+^ cells and TIM3 expression on CD8^+^ cells in mice treated with either IgG or anti‐TIM3 mAb. The quantification of CD4^+^ cells (C) and TIM3^+^ CD4^+^ cells (D) from spleen, draining lymph node (LN), blood, or tumor in each group of mice (mean ± SEM,* n *=* *6 mice, respectively, *t*‐test, *P <* *0.05, **P <* *0.01). The quantification of CD8^+^ cells (E) and TIM3^+^ CD8^+^ cells (F) from spleen, LN, blood, and tumor in each group of mice (mean ± SEM,* n *=* *6 mice, respectively, *t*‐test, *P <* *0.05, **P <* *0.01, ***P <* *0.001).

To explore the mechanism of antitumor activity of anti‐TIM3 therapy, we investigated the CD4^+^ T cells, CD8^+^ T cells, TIM3^+^CD4^+^ cells, and TIM3^+^CD8^+^ cells in both tumor microenvironment and peripheral environment, including spleen, draining LN, and blood of HNSCC mice as shown by representative pictures (Fig. 6A,B). TIM3 blockade increased the effector T cells, CD4^+^ and CD8^+^ T cells in tumor microenvironment and peripheral environment, especially in tumor and LN (Fig. 6C,E). Meanwhile, the TIM3 expression on CD4^+^ T cells and CD8^+^ T cells was down‐regulated by the blockade of TIM3, especially in tumor and LN (Fig. 6D,F), as the ratio of TIM3^+^ cells in CD4^+^ or CD8^+^ T cells was found to be decreased. These findings suggested that blockade of TIM3 could restore the effector T cells through modulating TIM3 expression on CD4^+^ or CD8^+^ T cells.

**Figure 7:**
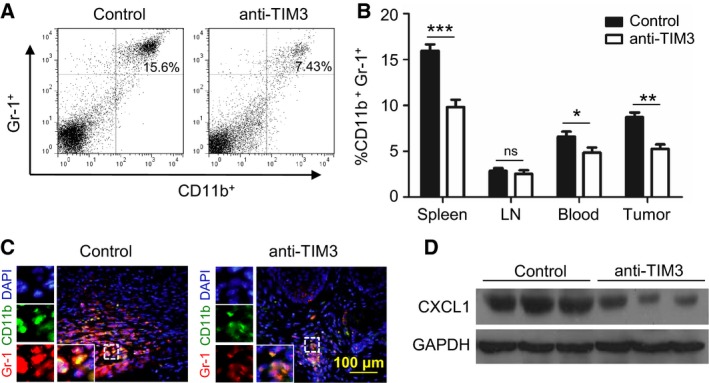
Blockade of TIM3 decreases MDSCs in HNSCC mouse model. (A) The representative FACS plots of CD11b^+^Gr‐1^+^ MDSCs in mice treated with either IgG (control) or anti‐TIM3 mAb. (B) Ratio of MDSCs from spleen, lymph node (LN), blood, or tumor in each group of mice (mean ± SEM,* n *=* *6 mice, respectively, *t*‐test, *P <* *0.05, **P <* *0.01; ***P <* *0.001). (C) Immunofluorescence of CD11b and Gr‐1 in tumor demonstrated that expression of CD11b and Gr‐1 is decreased in mice treated with anti‐TIM3 treatment (lower panel) as compared with that in mice treated with IgG (control, Left panel). (D) Representative western blot shows a decrease in CXCL1 expression in anti‐TIM3 mAb‐treated 2cKO mice HNSCC as compared with isotype IgG‐treated counterpart (control, *n* = 6 mice, respectively).

MDSCs are a diverse cellular population of myeloid origin with T‐cell‐suppressive functions (Huang *et al*., 2015). As shown in human HNSCC tissue array analysis, TIM3 was closely associated with MDSC markers, CD11b and CD33. To further determine the effect of anti‐TIM3 therapy on immune response in HNSCC mouse model, we investigated the CD11b^+^Gr1^+^ MDSCs from the spleen, LN, blood, and tumor tissue in HNSCC mice with or without anti‐TIM3 treatment (Fig. 7A). We found that population of MDSCs was significantly decreased by blockade of TIM3 (Fig. 7B). This finding was also confirmed by the immunofluorescence of CD11b and Gr1 (Fig. 7C). Further analysis of the tumor samples by western blot demonstrated that blockade of TIM3 attenuates the recruitment of MDSCs by reducing chemokine CXCL1 in tumor (Fig. 7D). The decrease in MDSCs also contributes to the restoration of effector T cells.

## 4 Discussion

TIM3, as a member of TIM family, has been identified as an important immune checkpoint inhibitor that plays an important role in modulating dysfunctional or exhausted CD8^+^ T cells in chronic diseases such as cancer (Sakuishi *et al*., 2013). Emerging evidence has demonstrated TIM3 functions in regulating immune response during cancer progression (Ngiow *et al*., 2011b; Zheng *et al*., 2015). However, its actual role in HNSCC has not been determined.

In this study, we demonstrated the increased expression of TIM3 in human HNSCC through IHC staining of HNSCC tissue. Nonetheless, the analysis of clinical data of HNSCC tissue suggested that TIM3 was not associated with pathological grades and TNM categories. Though, other reports have stated that TIM3 was positively correlated with overall survival in pancreatic ductal adenocarcinoma (Farren *et al*., 2016), and higher expression of TIM3 implicated in a worse 5‐year survival in renal cell carcinoma (Zheng *et al*., 2015). In the present study, based on the results of human HNSCC tissue and TCGA database, TIM3 expression was not correlated with overall survival of patients with HNSCC. This discrepancy of survival analysis may attribute to the difference in the pathophysiologic feature and TIM3 expression in different tumor types. Depending on the populations of T cells with distinct quantity and quality, immune system has the great potential for long‐term tumor control that can prevent cancer metastasis and recurrence. Our study demonstrated that increased TIM3 expression was correlated with LN metastasis and HNSCC recurrence. The exhaustion of effector T cells caused by the elevated TIM3 expression may lead to an invalid antitumor immune response and tumor elimination, which account for the metastasis and recurrence of HNSCC (Camus *et al*., 2009; Finn, 2012). Chemotherapeutics or radiotherapy is known to trigger immunogenic cell death (Apetoh *et al*., 2007; Welsh *et al*., 2014). Recent report indicated that radiotherapy could induce the increase in PD1^+^Tregs (Napolitano *et al*., 2015). In the present study, we found that TIM3 expression in HNSCC postradiotherapy and HNSCC post‐TPF chemotherapy was significantly higher than that in primary HNSCC, which indicates that TPF chemotherapy and radiotherapy induce the up‐regulation of TIM3.

TIM3 has been reported to be located in different leukocyte subsets (Ngiow *et al*., 2011a), and targeting TIM3 played an antitumor function (Ngiow *et al*., 2011b). In human HNSCC tissue, remarkable associations were found between TIM3 and CD8, CD33 and CD11b, which indicates the tight relation between TIM3 and effector T cells, MDSCs. To verify the role of TIM3 in HNSCC development, we utilized the *Tgfbr1/Pten* 2cKO mice in which TIM3 could be induced for spontaneous HNSCC formation (Bian *et al*., 2012). The prophylactic anti‐TIM3 treatment for HNSCC mice could suppress the tumorigenesis, and blockade of TIM3 notably improved immune response with increased populations of CD4^+^ and CD8^+^ T cells through modulating the CD4^+^TIM3^+^ cells and CD8^+^TIM3^+^ cells.

Inhibition of T cells is an important characteristic of MDSCs that contribute to immune suppression (Bronte *et al*., 2016; Cao *et al*., 2010); blockade of TIM3 decreases the CD11b^+^Gr‐1^+^ MDSCs by reducing CXCL1, which is an important chemokine for the recruitment of MDSCs (De Costa *et al*., 2012). From these findings, activation of TIM3 provided a mechanism of tumor evasion in HNSCC, and blockade of TIM3 reversed the immunosuppressive status by restoring the T‐cell activation and inhibiting MDSC aggregation.

In recent years, a variety of monoclonal antibodies has been approved by US Food and Drug Administration for use in patients with cancer (Vacchelli *et al*., 2014). So far, monoclonal Abs targeting PD‐1 or CTLA‐4 are being investigated in several clinical trials for different stages and disease status of patients with HNSCC (Ferris, 2015). Clinical trial of TIM3 is supported in phase I‐Ib/II study of the safety and efficacy of MBG453 as single agent and in combination with PDR001 in patients with advanced malignancies (NCT02608268). However, on‐target blockade of immune checkpoint inhibitor demonstrated limited efficiency for patients, because Hammerman and colleagues showed that TIM3 up‐regulated as a result of adaptive resistance for anti‐PD‐1 therapy (Koyama *et al*., 2016). Thus, combination therapy seems promising for cancer therapy (Sathyanarayanan and Neelapu, 2015), as immunotherapy targeting TIM3 appeared to be effective in combination with anti‐PD‐1 or anti‐CTLA‐4 (Hervas‐Stubbs *et al*., 2016; Ngiow *et al*., 2011b). The current study suggests that blockade of TIM3 has important therapeutic implications for the treatment of HNSCC.

In summary, we demonstrated the TIM3 expression in HNSCC, which is closely associated with immune effector CD8^+^ T cells and important immune suppressor MDSCs. Taking advantage of immunocompetent *Tgfbr1/Pten*‐knockout HNSCC models, we observed restoration of effector T cells by targeting CD4^+^TIM3^+^ cells and CD8^+^TIM3^+^ cells and decreasing MDSCs in tumor microenvironment and peripheral environment. Therefore, we believe that blockade of TIM3 will be a promising antitumor therapy for HNSCC.

## Author contributions

JFL, WFZ, and ZJS conceived and designed the project. JFL, SRM, GTY, LM, and WWD acquired the data. JFL, LLB, YCL, and CFH analyzed and interpreted the data. JFL, ABK, and ZJS wrote the paper.

## Supporting information


**Fig. S1.** *HAVCR2* (encoding TIM3) is overexpressed in human HNSCC.
**Table S1.** Clinicopathological statistics of HNSCC used in this study.
