# Supplementary material for: T‐cell immunoglobulin mucin 3 blockade drives an antitumor immune response in head and neck cancer
Source: Mol Oncol. 2017 Jan 19;11(2):235–47. doi: 10.1002/1878-0261.12029 (PMC5527458; doi:10.1002/1878-0261.12029)
Supplement: Supplementary file 1 — Fig. S1. HAVCR2 (encoding TIM3) is overexpressed in human HNSCC. Table S1. Clinicopathological statistics of HNSCC used in this study. [file MOL2-11-235-s001.pdf]

Supplementary Materials

A

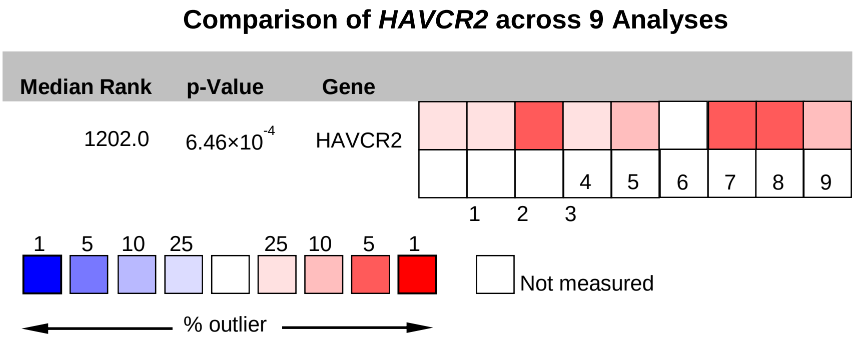

Legend

1. Head and Neck Cancer vs. Normal
   Barretina CellLine 2, Nature, 2012
2. Head and Neck Cancer vs. Normal
   Beroukhim Multi-cancer, Nature, 2010
3. Oral Cavity Squamous Cell Carcinoma vs. Normal
   Peng Head-Neck, PLOS One, 2011
4. Floor of the Mouth Carcinoma vs. Normal
   Pyeon Multi-cancer, Cancer Res, 2007
5. Oropharyngeal Carcinoma vs. Normal
   Pyeon Multi-cancer, Cancer Res, 2007
6. Tongue Carcinoma vs. Normal
   Pyeon Multi-cancer, Cancer Res, 2007
7. Head and Neck Squamous Cell Carcinoma vs. Normal
   Rickman Head-Neck, Oncogene, 2008
8. Head and Neck Squamous Cell Carcinoma vs. Normal
   Slebos Head-Neck, Clin Cancer Res, 2006
9. Head and Neck Cancer vs. Normal
   Wooster CellLine 2, Not Published, 2008

B

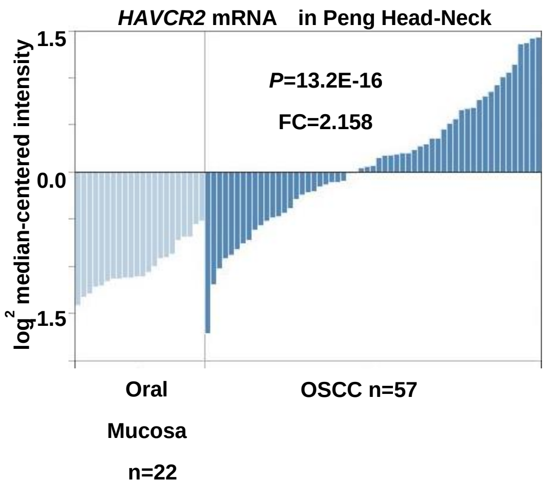

Supplementary Fig. 1 *HAVCR2* (encoding TIM3) is over-expressed in human HNSCC. (A) meta-analysis of gene expression profiling for *HAVCR2* in human HNSCC. The colored squares indicated the median rank for *HAVCR2* across each analysis. Data

retrieve from Oncomine database. (B) Significant increase of *HAVCR2* from Peng's dataset.

Supplementary Table 1. Clinicopathological statistics of HNSCC used in this study.

**Parameters**	**No.**
**Normal Mucosa(Muc)**	27
**Dysplasia(Dys)**	43
**Primary HNSCC(PH)**	122
**Grade**	
**I**	35
**II**	65
**III**	22
**Lymph node metastasis(negative)**	78
**Lymph node metastasis(negative)**	44
**Recurrent HNSCC(RH)**	8
**HNSCC with pre-radiotherapy (RT)**	12
**HNSCC with pre-chemotherapy(TPF)**	11
